# Quantifying Shark Distribution Patterns and Species-Habitat Associations: Implications of Marine Park Zoning

**DOI:** 10.1371/journal.pone.0106885

**Published:** 2014-09-10

**Authors:** Mario Espinoza, Mike Cappo, Michelle R. Heupel, Andrew J. Tobin, Colin A. Simpfendorfer

**Affiliations:** 1 Centre for Sustainable Tropical Fisheries and Aquaculture and School of Earth and Environmental Sciences, James Cook University, Townsville, Queensland, Australia; 2 AIMS@JCU, Australian Institute of Marine Science, School of Earth and Environmental Sciences, James Cook University, Townsville, Queensland, Australia; 3 Australian Institute of Marine Science, Townsville, Queensland, Australia; The Australian National University, Australia

## Abstract

Quantifying shark distribution patterns and species-specific habitat associations in response to geographic and environmental drivers is critical to assessing risk of exposure to fishing, habitat degradation, and the effects of climate change. The present study examined shark distribution patterns, species-habitat associations, and marine reserve use with baited remote underwater video stations (BRUVS) along the entire Great Barrier Reef Marine Park (GBRMP) over a ten year period. Overall, 21 species of sharks from five families and two orders were recorded. Grey reef *Carcharhinus amblyrhynchos*, silvertip *C. albimarginatus*, tiger *Galeocerdo cuvier*, and sliteye *Loxodon macrorhinus* sharks were the most abundant species (>64% of shark abundances). Multivariate regression trees showed that hard coral cover produced the primary split separating shark assemblages. Four indicator species had consistently higher abundances and contributed to explaining most of the differences in shark assemblages: *C. amblyrhynchos*, *C. albimarginatus*, *G. cuvier*, and whitetip reef *Triaenodon obesus* sharks. Relative distance along the GBRMP had the greatest influence on shark occurrence and species richness, which increased at both ends of the sampling range (southern and northern sites) relative to intermediate latitudes. Hard coral cover and distance across the shelf were also important predictors of shark distribution. The relative abundance of sharks was significantly higher in non-fished sites, highlighting the conservation value and benefits of the GBRMP zoning. However, our results also showed that hard coral cover had a large effect on the abundance of reef-associated shark species, indicating that coral reef health may be important for the success of marine protected areas. Therefore, understanding shark distribution patterns, species-habitat associations, and the drivers responsible for those patterns is essential for developing sound management and conservation approaches.

## Introduction

Predicting shark occurrences and species-specific habitat associations in response to geographic, habitat and environmental drivers can be a powerful approach in regional conservation planning [1]. Distribution patterns of shark biodiversity are generally associated with latitudinal and bathymetric gradients [2], [3]. Shark species richness typically increases toward the equator and peaks in shallow continental shelf waters (<200 m), where approximately 41% of all species occur [2], [4]. However, the drivers responsible for shark occurrences and species-habitat associations can vary considerably between regions and are often poorly understood. While some species exhibit a strong association with particular habitats (i.e. coral reefs) [5]–[7], in general, most sharks tend to use a wide variety of habitats along the continental shelf [8]–[11], potentially acting as energy links in the transfer of nutrients from one system to another [12]. Therefore, understanding species-specific habitat associations over large spatial scales can be a valuable approach to identify important areas for shark conservation, as well as elucidate complex ecological processes such as connectivity within and across ecosystems.

The Great Barrier Reef (GBR) is one of the most productive and globally important hot spots of marine biodiversity [4], [13]. Within the GBR, elasmobranchs comprise a highly diverse group (134 species from 41 families) characterized by a wide range of life-history strategies [14] and varying degrees of vulnerability to both climate and anthropogenic pressures [8], [11], [15]. Sharks represent approximately 60% of the GBR's elasmobranch diversity and are thought to play a key role in the structure and functioning of marine communities through “top down” predation pressure on lower trophic levels [16], [17]. However, several shark species are subject to fishing pressure (e.g. some species are taken intentionally, or as bycatch, in a variety of fisheries), which in some cases has resulted in significant declines in the abundance of reef sharks [18]–[20]. Moreover, increased frequency of disturbances and anthropogenic activities within the GBR are having a major impact on coral reefs [21], [22], and ultimately on reef-associated sharks. Therefore, knowledge of shark species ranges and habitat associations along the GBR must be understood to assess the risk of exposure to fishing, habitat degradation and the effects of climate change [15], [23].

The GBR has the largest and most intensively managed network of Marine Protected Areas (MPAs) in the world, ranging from open-access (areas open to all human activities) to no-entry [24], [25]. Approximately 33% of the GBRMP has been designated as no-take zones (areas closed to all forms of fishing), providing protection to a range of bioregions [24]. Marine reserve networks such as the GBRMP are thought to offer greater protection for mobile species by reducing their exposure to fisheries [25], [26]. Although the benefits of MPAs for individual shark species have been poorly documented [5], [27]–[29], a variety of models and empirical studies suggest that spatial management approaches are critical for shark conservation [5], [30], and ultimately may help maintain ecosystem resilience [31], [32].

The use of fish habitats and species assemblages as surrogates for biological diversity is becoming increasingly popular in spatial planning [33], [34]. Baited remote underwater video stations (BRUVS) have been previously used to document fish species richness along geographic gradients [33], [35], quantify elasmobranch abundances and distribution patterns [5], [36], understand biases of sampling gears [37], [38], and compare fish densities inside and outside marine reserves [32], [39]. Therefore, BRUVS may provide a “non-destructive/non-extractive” approach for quantifying shark occurrences and documenting species-habitat associations over large spatial scales. The present study examined shark distribution patterns, species-habitat associations and marine reserve use with BRUVS along the entire GBRMP over a ten year period. Multivariate prediction and regression trees were used to identify shark assemblages and examine species-specific associations in relation to depth, habitat cover, geographic (relative distance along/across the shelf, reef proximity), and environmental (sea surface temperature and chlorophyll-a) drivers. The effects of zoning (e.g. areas open and closed to fishing), habitat and time since the 2004 re-zoning of the GBRMP on shark abundances were examined using Poisson and Negative Binomial regression models.

## Methods

### Study area and sampling design

**Figure 1:**
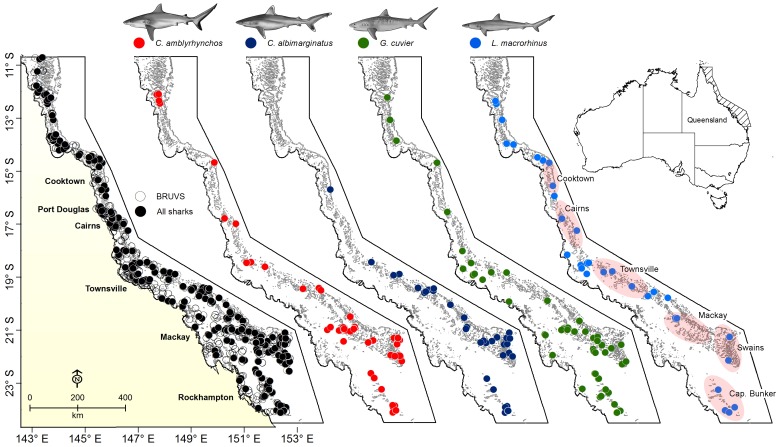
Map of the Great Barrier Reef Marine Park (Australia) showing the location of all baited remote underwater video stations sampling sites and the distribution of sightings for the most common sharks.

The GBRMP is characterized by a wide range of habitats, including coral reefs, mangrove/estuaries, sandy bays, seagrass beds, soft-sediment inter-reef habitats, and rocky shoals dominated by diverse groups of octocorals (e.g. soft corals, sea fans, sea pens) [40], [41]. The GBRMP has approximately 3,000 reefs distributed over 2,300 km (between 11° and 25°S) and an area of approximately 348,000 km^2^ (Fig 1). Most reefs (∼2,400) are located offshore on the mid- and outer-continental shelf; the rest (∼600) are located inshore, either as fringing reefs (around continental islands and along the coastline) or as small, isolated patches or platform reefs [42]. The present study analysed a historical collection of data from biodiversity surveys conducted between 2000 and 2010 in the GBRMP using BRUVS [35], [37]. Biologically informed stratification was used to sample a wide range of habitat types (e.g. reef, inter-reef, shoal and lagoonal habitats) of the GBRMP. A total of 2471 BRUVS were deployed between March 2000 and May 2010 covering the entire range of latitudes (10.7°S–24.2°S) and longitudes (143.38°E–152.36°E) of the GBR [35], [37]. BRUVS were deployed at depths of 7 to 115 m (mean ±SD; 36.7±15.6 m) and set approximately 350–400 m apart. Most stations were deployed during day-times, but a small sample of BRUVS (<2%) were night-time sets [37]. Both day and night-time sets were pooled and analysed together. Water visibility varied between 0.5 and 20 m, with a mean ±SD visibility of 6.7±3.8 m. For a detailed description of environmental conditions, including visibility recorded during BRUVS deployments see Dataset S1.

A roll-cage frame was used prior to 2003 [37], and a trestle-shaped frame was used afterward, for the majority of BRUVS deployments (Fig. S1a). A simple camera housing made from PVC pipe with acrylic front and rear ports was used inside the frames to deploy either a Sony Hi-8 (model TR516E; prior to 2003) or a Sony Mini-DV (models TRV18E, TRV19E) HandiCam. Exposure was set to “Auto”, focus was set to “Infinity/Manual”, and “Standard Play” mode was selected to provide at least 45 min of filming at the seabed (mean ±SD; 53.3±11.3 min). Detachable bait arms (20 mm plastic conduit) had a 350 mm plastic mesh canister containing 1 kg of crushed oily sardines (*Sardinops* or *Sardinella* spp.) as bait, lying on the seabed. BRUVS were deployed with 8 mm polypropylene ropes and polystyrene surface floats bearing a marker flag, and were retrieved with hydraulic pot-hauler wheel [35], [37]. Each BRUVS video tape was examined using a custom interface (BRUVS1.5.mdb, Australian Institute of Marine Science, 2006): 1) to manage data from field operations and tape reading; 2) to capture the timing of events; and 3) to capture reference images of the seafloor and sharks in the field of view. The maximum number of individuals from each shark species observed together in any one time on the whole tape was recorded as MaxN [37]. Species were identified to the lowest taxonomic level possible by analyzing the collection of reference images with shark specialists (see Fig. S1b,c,d). Unidentified species (<5% of all records) were pooled at the genus level. Hereafter, these taxa are referred to as species. Shark species were classified as juveniles and adults based on length measurement analysis of video tape readings.

### Habitat classification and environmental drivers

The date/time, location (latitude/longitude), depth (m), soak time (hrs) and distance to the nearest reef feature (km) were recorded for each BRUVS. Calculations of distance from the nearest reef were made for each deployment using spatial data layers from the GBR Marine Park Authority website (http://www.gbrmpa.gov.au/). To improve the analysis and interpretation of the spatial distribution patterns of sharks, latitude and longitude were converted into a relative distance index “across” and “along” the GBRMP [43]. Relative distance across was set to 0 on the coast and 1on the outermost edge of the continental shelf (80 m isobath), and distance along the shelf ranged from 0 on the southern edge of the GBR to 1 on the northern edge [43]. Benthic habitat characterization was possible by analyzing the collection of reference images. For each image, two independent observers qualitatively estimated the percent cover of six major benthic groups: 1) plants/macro algae; 2) soft coral; 3) hard coral; 4) other filter-feeders (e.g. sponges, clams); 5) bare sand/mud; and 6) encrusting algae/rubble. Conflicting estimates of percent cover between observers were presented as a mean of the two estimates. A qualitative index (1–4; low to high) was used to assess the degree of topographic complexity of the seafloor for each image. Shark distribution patterns in relation to monthly daytime (4 km) chlorophyll-a (mg C m^−2^ day^−1^) and sea surface temperature (°C) were examined by consulting available remote sensing data for the GBRMP (http://www.oceancolor.gsfc.nasa.gov/; http://www.nodc.noaa.gov/SatelliteData). Seasons were defined as summer (December-February), autumn (March-May), winter (June-August) and spring (September-November).

### Data analysis

The BRUVS dataset used here was not collected specifically to examine shark distribution patterns. Throughout this survey, some locations were sampled more intensively than others to answer specific questions. To avoid any potential sampling bias, the dataset was analysed in two ways: 1) at the BRUVS level (2,438 unique BRUVS); and 2) at the site level (590 unique sites). At the BRUVS level, a principal component analysis (PCA) was performed by constraining the BRUVS scores to display only the variation among BRUVS that could be explained by the percent cover of major habitat types [44]. This reduced the number of habitat components that explained >96% of the variability amongst BRUVS into three major principal scores: 1) bare to cover (PC1); 2) algae/plants to rubble (PC2); and 3) algae/plants to coral cover (PC3) (Table S1). Sites were defined based on the location (stations that were <1 km apart) and date of each station. Stations that were deployed at the same site but on different dates were considered independent samples. Replicate MaxN of each shark species were summed across sites. To standardize the sampling effort, the total hours of video (soak time) were summed for each site. Relative abundance was defined as the total MaxN of each species per site divided by the effort (MaxN hrs^−1^). Cumulative species richness curves were examined at the BRUVS and site level. The order in which shark species were analysed was randomized 999 times and the cumulative number of new species per station/site was counted for each randomization. Subsequently, the number of BRUVS and sites were plotted against the mean ±SD number of species.

Shark community composition was determined with multivariate regression trees (MRT) using presence-absence data at the site level [45]. Only species that were sighted on over 5% of the sites were included: grey reef *Carcharhinus amblyrhynchos*, tiger *Galeocerdo cuvier*, silvertip *C. albimarginatus*, sliteye *Loxodon macrorhinus*, tawny nurse *Nebrius ferrugineus*, great hammerhead *Sphyrna mokarran* and whitetip reef *Triaenodon obesus* sharks. The mean and standard deviation of predictor variables (e.g. habitat and environmental drivers) used in the MRT analysis were calculated for each site and used as predictors in the models. The nodes of the MRT define a hierarchy of maximal dissimilarity assemblages characterized by distinct spatial-environment associations. Cross-validation was used to identify the size of the tree that minimized prediction error [45]. For interpretation of the MRT, the Dufrêne-Legendre indicator value (DLI) of each species was estimated at each node of the tree [46]. The DLI value for a given species in assemblage A was defined as: *DLI_A_ = 100× (P_A_)^2^/ΣP_A_*, where PA represents the proportion of BRUVS/sites in assemblage A where the species is present, *Σ* indicates summation over all the assemblages [41], [46]. The DLI values can range from 0 (no occurrence of a species at any BRUVS/site of an assemblage) to 100 (the species occurs at all sites in the assemblage and nowhere else). Each species was associated with the node of the tree where it had the maximum DLI value. High DLI values (>20) were used to define indicators of species assemblages and the relative importance of predictor variables that explained their occurrences.

Shark species richness, and the occurrence of indicator species identified by MRT (species with DLI values >20: *C. amblyrhynchos*, *C. albimarginatus*, *G. cuvier* and *T. obesus*), were both analysed using aggregated boosted regression trees (ABT) at the site level [47]. Boosted trees are a regression and classification technique based on adaptive learning, which can be used to examine detailed species-environment relationships [48]. The ABTs are an extension of boosted trees that improve the predictive performance of boosted regression trees [47]. The following predictors (mean ±SD values per site) were used in the model: depth, relative distances across and along the shelf, distance to nearest reef feature (km), SST (°C), chlorophyll-a concentration (mg C m^−2^ day^−1^), complexity index, and the PC habitat scores (e.g. PC1: bare-cover, PC2: algae/plants-rubble and PC3: algae/plants-coral). ABT models included all predictors and up to third order interactions, and monotonic constraints were applied to the functional form of selected predictors [47]. Cross-validation of the ABTs based on site was used to select the best predictive model. Models were compared using: 1) the mean square predictive error for each model expressed as a percentage of the variance of the response variable (%PE); 2) the importance of each predictor estimated as the percentage of variation explained; and 3) partial dependency plots to illustrate the relationship between species richness and occurrence of the most common sharks and the predictors. All analyses were done using R statistical package v.3.0.2, including the libraries mvpart for multivariate trees and abt/gbm for boosted trees [49].

The effect of the zoning on abundance was evaluated at the site level. Spatial layers for the GBRMP's official zoning (before and after the 2004 re-zoning) were obtained from the GBRMPA website (http://www.gbrmpa.gov.au/). Zoning was classified into: 1) areas closed to all forms of fishing, including no-take and no-entry zones; and 2) areas open to different levels and methods of fishing, including recreational and commercial. Most sites surveyed before 2006 were in areas open to fishing (72.1%), whereas only 1.9% of sites during the same period were closed to fishing. Sampling effort between 2006 and 2010 was similar in areas closed (14.5%) and open (11.5%) to fishing. Therefore, only sites surveyed between 2006 and 2010 were used in the analysis. Additionally, most of these sites (97%) used in the analysis were open to fishing since the creation of the GBRMP. This facilitated comparison and interpretation of the results, and also avoided the risk of introducing a confounding factor by comparing sites that were recently closed to fishing with those that had been closed since the creation of the GBRMP and are presumably healthier.

Negative binomial and Poisson general linear modelling were used to examine the effect of zoning (e.g. areas closed/open to fishing) and habitat (hard coral cover and reef proximity) on shark abundance. In cases of over dispersion in count data, negative binomial (NB) models typically performed better than Poisson (P) [50]. Several models were examined using the relative abundance data (MaxN hr^−1^) of: 1) all shark species combined; 2) *C. amblyrhynchos*; 3) *C. albimarginatus*; and 4) *G. cuvier* as the response variables. These species had a MaxN >40 and were observed on at least 15% of the sites. The number of days since the GBR re-zoning (effective since July 2004) and habitat (e.g. the percent of hard coral and proximity to reef features) were also included as continuous covariates to examine their effect on shark abundance. Sampling effort (hrs) was included as an offset to account for variability in hours of footage at each site. The performance of P and NB models were compared using maximum likelihood ratio tests and Akaike's information criterion (AIC) of nested models. To examine the importance of variables used in the models, the difference in AIC with and without each term was computed using likelihood ratio tests. These analyses were done using the libraries pscl, MuMIn and lmtest from R statistical package v.3.0.2 [49].

## Results

**Figure 2:**
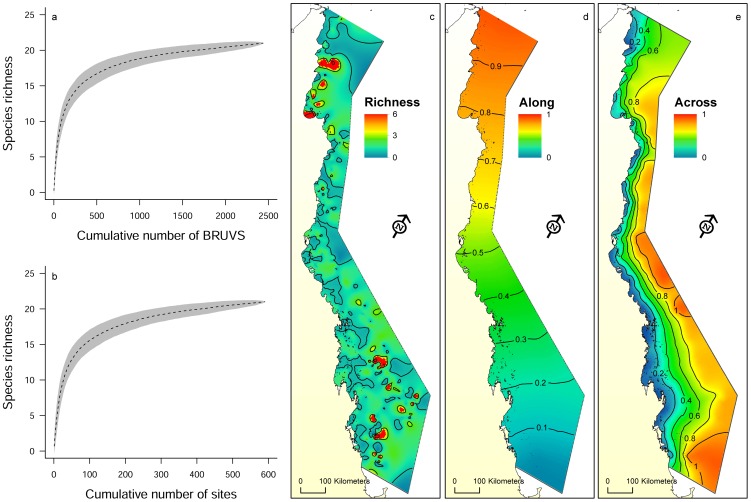
Shark species richness (mean ±SD) by (a) the cumulative number of baited remote underwater video stations and (b) the cumulative number of sites surveyed. Maps show the distribution of shark species richness (c), and patterns (contours and colour shading) of variation of location along (d) and across (e) the Great Barrier Reef (GBR) continental shelf (rotated view), using an interpolation with a smooth spline with barriers technique. Distance along the shelf ranged from 0 on the southern edge of the GBR to 1 on the northern edge. Distance across was set to 0 on the coast and 1on the outermost edge of the continental shelf (80 m isobath).

Distribution and shark species richness was examined at 2438 BRUVS and 590 sites (Fig 1). Although most BRUVS were deployed at inter-reef habitats dominated by soft sediments, they were in close proximity to coral reefs (0–45.4 km). Average SST and chlorophyll-a concentration for the sampling period showed little variability (Table 1). Overall, sharks were rare or uncommon, occurring in approximately 25% of the BRUVS examined. Sharks were sighted at 614 stations and the number of sightings per BRUVS varied between 1 and 7 sharks (mean ±SD: 1.31±0.71). Species richness varied between 1 and 3 (mean ±SD: 1.14±0.38), with most BRUVS where sharks were sighted (87%) recording a single species. Sharks were sighted at 271 sites (46% of sites), and the number of sightings per site varied between 1 and 39 sharks (mean ±SD: 1.36±3.15). Species richness per site varied between 1 and 7 (mean ±SD: 1.62±0.98), with 61% and 24% of sites containing one and two species, respectively. Cumulative species curves at the BRUVS (Fig 2a) and site levels (Fig. 2b) revealed that enough sampling units were examined to accurately describe shark assemblages.

**Table 1:** Predictors used in the aggregated boosted regression tree and multivariate regression tree analyses.

Predictor	Type	Range	Mean ±SD
Depth (m)	Continuous	7–104	38.04±16.91
Along	Continuous	0.02–1.0	0.41±0.24
Across	Continuous	0.02–1.0	0.48±0.27
Nearest distance to reef edge (km)	Continuous	0–45.22	6.56±7.58
SST (°C)	Continuous	20.55–30.38	25.91±2.34
Chlorophyll-a (mg C m^−2^ day^−1^)	Continuous	0.10–2.82	0.48±0.43
Complexity index	Categorical	1–4	1.37±0.55
Season	Categorical	NA	NA
% Marine plants / macro algae	Continuous	0–100	8.0±14.86
% Soft coral	Continuous	0–30	2.67±4.95
%Hard coral	Continuous	0–63.33	3.14±7.93
% Filter-feeders	Continuous	0–10	0.43±1.47
% Bare sand/mud	Continuous	0–100	74.28±27.45
% Encrusting algae/rubble	Continuous	0–100	11.49±14.65
PC1: bare to cover	Continuous	−2.20–0.88	0.17±0.75
PC2: algae/plants to rubble	Continuous	−3.29–4.34	0.03±0.78
PC3: algae/plants to coral cover	Continuous	−3.61–2.76	−0.10±0.69

Most sites surveyed had relatively few shark species (1–2 species) occurring together, particularly in the central GBR along coastal bays and inter-reef waters. However, some sites had disproportionally higher shark species richness. For example, 28 of the 36 sites that had three or more shark species were located in the southern GBR (e.g. Mackay, Swains and Capricorn Bunker group). In the northern GBR (north of Cooktown), seven sites had three or more shark species, while in the central GBR (e.g. Cairns and Townsville) only one site had high species richness (Fig. 2c). A total of 21 species of sharks from five families and two orders were documented (Table 2). Eleven species had a MaxN greater than 20 and accounted for over 92% of the total shark abundance (total MaxN  = 804). *Carcharhinus amblyrhynchos*, *C. albimarginatus*, *G. cuvier*, *L. macrorhinus*, *N. ferrugineus* and *T. obesus* were the most sighted species and represented over 76% of the total shark abundance (Table 2, Fig. 1). In addition, with the exception of Australian blacktip sharks *C. tilstoni*/C. *limbatus* (69.6% juveniles) most sharks sighted were classified as adults.

**Table 2:** Summary of shark sightings, abundance (MaxN; % MaxN) and the proportion of adults recorded on baited remote underwater video stations.

Family	Species	No. Sightings	MaxN	MaxN (%)	Adults (%)	MaxN Closed	MaxN Open	Habitat	Depth (m)	Along	Across
Carcharhinidae	*Carcharhinus albimarginatus*	87	98	12.2	73.6	60	38	SH-CR	21.2–76.1	0.03–0.68	0.50–1.0
	*Carcharhinus amblyrhynchoides*	7	8	1.0	71.4	0	8	CR	19.6–32.3	0.55–0.82	0.13–0.48
	*Carcharhinus amblyrhynchos*	184	247	30.7	83.7	172	75	CR	14–72.1	0.03–0.92	0.30–0.1
	*Carcharhinus amboinensis*	1	1	0.1	100	0	1	IN-SH	37.4–37.4	0.74	0.81
	*Carcharhinus brevipinna*	1	1	0.1	100	1	0	IN-SH	40.7–40.7	0.05	0.80
	*Carcharhinus coatesi*	23	23	2.9	91.3	2	21	IN	14.7–58.6	0.08–0.89	0.06–0.43
	*Carcharhinus leucas*	4	4	0.5	100	0	4	IN-SH-CR	17.7–34.7	0.03–0.61	0.33–0.62
	*Carcharhinus melanopterus*	1	1	0.1	100	1	0	IN-CR	40.0	0.46	0.72
	*Carcharhinus plumbeus*	24	25	3.1	91.7	4	21	SH	19.7–74.9	0.03–0.45	0.19–1.0
	*Carcharhinus* sp.	11	11	1.4	63.6	1	10	IN-SH	20–40	0.50–0.81	0.18–0.57
	*Carcharhinus tilstoni/limbatus*	23	24	3.0	30.4	1	23	IN-SH	16.9–76.9	0.10–1.0	0.04–0.71
	*Galeocerdo cuvier*	94	97	12.1	75.5	36	61	IN-SH-CR	15.3–85	0.03–0.91	0.06–1.0
	*Loxodon macrorhinus*	56	75	9.3	92.9	3	72	IN-SH-CR	30.7–38	0.03–0.90	0.28–1.0
	*Negaprion acutidens*	5	5	0.6	100	2	3	CR	15.8–53.8	0.03–0.91	0.31–0.79
	*Rhizoprionodon taylori*	19	20	2.5	100	2	18	IN-SH	34.7–50.6	0.05–0.93	0.06–0.83
	*Triaenodon obesus*	45	46	5.7	100	29	17	CR	16.8–80.7	0.03–0.75	0.29–0.94
Ginglymostomatidae	*Nebrius ferrugineus*	49	49	6.1	100	10	39	CR	12.8–74.6	0.03–0.99	0.04–1.0
Hemigaleidae	*Hemigaleus australensis*	4	4	0.5	100	1	3	IN-SH	30.7–50.6	0.08–0.53	0.62–0.85
	*Hemipristis elongatus*	6	6	0.7	100	3	3	IN-SH	18.9–42.4	0.19–0.54	0.13–0.74
Orectolobidae	*Orectolobus maculatus*	5	5	0.6	80.0	4	1	CR	17.4–52.2	0.03	0.62–0.8
Sphyrnidae	*Sphyrna lewini*	12	12	1.5	66.7	1	11	IN-SH-CR	17–74.6	0.13–1.0	0.04–0.49
	*Sphyrna mokarran*	41	41	5.1	97.6	14	27	IN-SH-CR	16.3–84.2	0.03–0.91	0.021–0.94
	*Sphyrna* sp.	1	1	0.1	100	0	1	IN-SH-CR	51.4–51.4	0.13–0.13	0.41

The MaxN recorded in areas open and closed to fishing is also presented. The depth and relative distances along and across the shelf where each species was sighted are presented as a range. MaxN: the maximum number of individuals from each species observed together in any one time on the whole tape; % MaxN: defined as the MaxN of each individual species divided by the total MaxN. Distance along the shelf ranged from 0 on the southern edge of the GBR to 1 on the northern edge. Distance across was set to 0 on the coast and 1on the outermost edge of the continental shelf (80 m isobath). Habitat type: SH - shelf; CR - coral reef; IN – inshore/coastal.

### Shark assemblage structure

**Figure 3:**
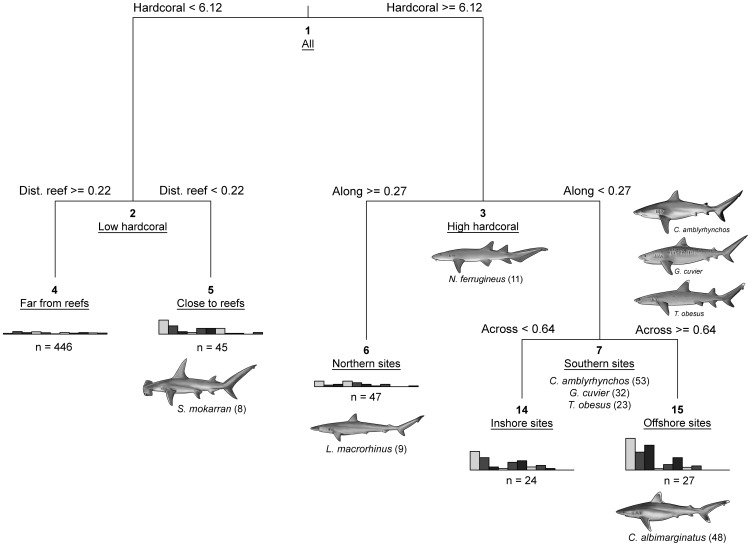
Multivariate regression tree analysis of the occurrence of shark species explained by 12 environmental/habitat predictors (Cross-Validated Error: 0.90±0.05 SE). The bold numbers at each node show the predictors that were most influential in predicting different shark assemblages. Histograms on the “leaves” show the frequency of occurrence of each species and the number of sites (n) with the node names and node numbers. The Dufrêne-Legendre species indicators (DLI) characterising each branch and terminal node (leaf) of the tree were included. Shark species at node 5: *Sphyrna mokarran*; node 6: *Loxodon macrorhinus*; node 7: *Carcharhinus amblyrhynchos, Galeocerdo cuvier, Triaenodon obesus*; node 15: *C. albimarginatus.*

Multivariate regression trees (MRTs) were used to investigate the hierarchical assemblage structure of sharks along the GBR. A tree with five terminal nodes was selected to represent the most parsimonious assemblage (Fig. 3). The MRT showed that the hard coral cover (≥6.12%) in the field of view was the primary predictor separating shark assemblages that occurred at sites with low and high coral cover. *Nebrius ferrugineus* was identified as an indicator species of “root node 3”, suggesting this species is common in habitats with high coral cover throughout the GBR and is not confined to a particular assemblage (terminal node, or “leaf”). Reef proximity was used for the second split, which separated reef sites (<220 m) from sites that were farther from reefs (≥220 m). The great hammerhead shark *Sphyrna mokarran* was identified as an indicator species of the assemblage that occurs at sites with low hard coral cover and close proximity to reefs (node 5, Fig. 3). The third split was the relative distance along the GBR, which separated the southernmost sites near the Swains Reefs (offshore) and Gladstone (inshore). The sliteye shark *Loxodon macrorhinus* was an indicator species for northern sites with high coral cover, whereas *C. amblyrhynchos*, *C. albimarginatus*, *G. cuvier* and *T. obesus* characterized the assemblages in southern sites with high coral cover (relative distance along <0.27; 22–24°S). The final split was between southern inshore and offshore sites (Fig. 3). *Carcharhinus albimarginatus* was the main indicator species driving the assemblage at southern offshore sites (>0.64). Overall, most species that occurred in each group were rare, but four species had consistently higher abundances and DLI values ≥20: *C. amblyrhynchos*, *C. albimarginatus*, *G. cuvier* and *T. obesus* and contributed to explaining most patterns in overall assemblages. The remaining species (80%) had low DLI values (5–15), and had both low abundances and occurrences at examined sites.

### Shark species-specific habitat associations

**Figure 4:**
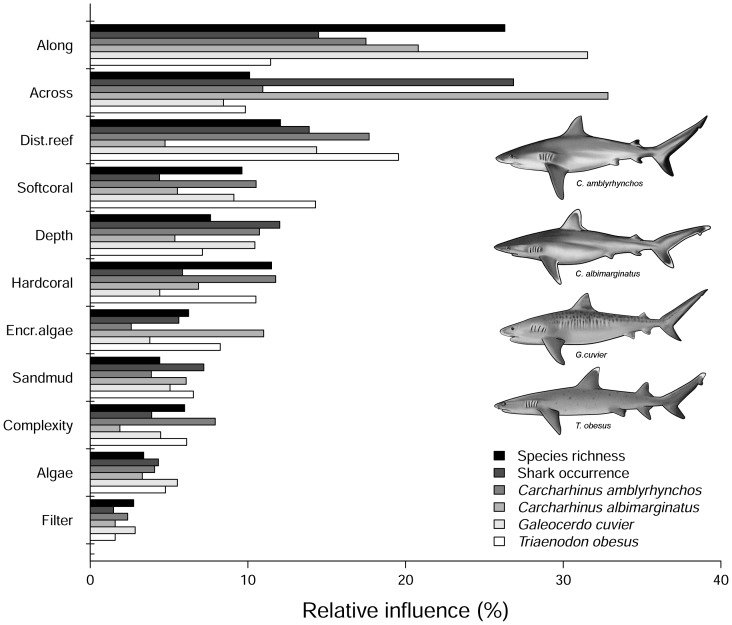
Summary of the relative contributions (%) of the top eleven predictors used in aggregated boosted regression trees (ABT). Models were developed with cross-validation on data from 364 sites using tree complexity of 5 and learning rate of 0.001. Shark species richness and the occurrence (presence-absence data) from the indicator species of shark assemblages (see Fig. 4) were used in the ABT.

**Figure 5:**
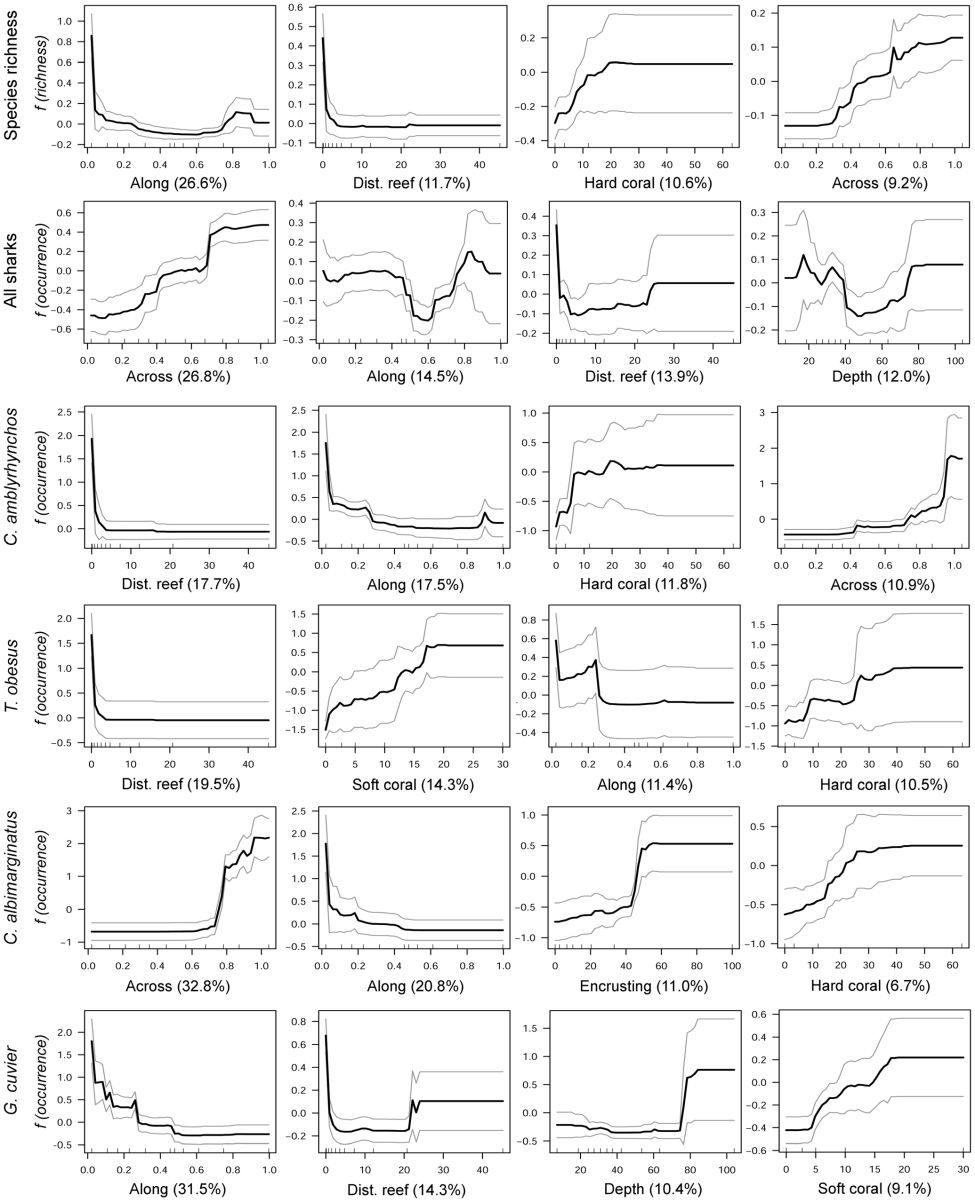
Partial dependency plots from the aggregated boosted regression tree analysis of the occurrence and richness of shark species observed on baited remote underwater video stations. The effects of the four most influential environmental/habitat predictors on the occurrence of *Carcharhinus amblyrhynchos*, *C. albimarginatus*, *Galeocerdo cuvier* and *Triaenodon obesus*. The bottom panel shows the effect of environmental predictors on species richness. For individual shark species, the y-axis represents the mean probability of occurrence centered at zero across all sites. Grey lines indicate ±2 SE for the predicted values, estimated from predictions made from 500 trees fitted in 5-fold cross validation at the site level.

Aggregated boosted tree analyses (ABT) showed that the relative distance “along” the GBR had the greatest influence on shark species richness (Fig. 4, Fig 5). Species richness increased at southern and northern sites and gradually decreased at intermediate latitudes (Fig. 5). The nearest distance to reef habitats and the percent of hard coral cover (combined relative influence: 23.6%) were also important in predicting shark species richness, which increased in response to proximity to reefs and coral cover. Sites with greater structural complexity (e.g. rocky shoals, coral reef environments, and habitats dominated by macro algae and marine plants), particularly on the outer half of the shelf (relative distance “across” >0.6) also had more species of sharks than coastal inshore habitats with lower complexity. The probability of shark occurrence along the GBR was influenced primarily by the relative distance across the shelf (Fig. 4). Shark sightings were more common at offshore sites than at inshore coastal habitats (Fig. 5). Additionally, the probability of shark sightings decreased at intermediate latitudes (distance along the GBR: 0.5–0.7; between Townsville and Cairns) and increased with reef proximity (combined relative influence >28%).

The relative influence of environmental/habitat predictors were examined for a subset of indicator species with DLI values >20: *C. amblyrhynchos*, *T. obesus*, *C. albimarginatus* and *G. cuvier*. Although these species were influenced in different ways by the predictors used in ABT analyses, relative distance along and across the shelf, reef proximity and percent of hard coral were consistently identified as the best predictors for their occurrence (Fig. 4, Fig. 5). Distance to reefs and relative distance along the GBR were the most influential predictors for the occurrence of *C. amblyrhynchos*, with a combined relative influence of over 35% (Fig. 5). Additionally, *C. amblyrhynchos* had a higher probability of occurrence in structurally complex habitats near hard substrata (e.g. rocky shoals, coral reefs, etc.). Sightings of *C. amblyrhynchos* were more likely at offshore sites (relative distance across >0.8), particularly in the southern GBR (Fig. 1, Fig. 5). Relative distance across the shelf explained 33% of the occurrence of *C. albimarginatus*, which was nearly absent from inshore sites (relative distance across <0.6). Probability of occurrence increased two-fold at sites on the outer shelf (relative distance across >0.8) (Fig 1, Fig. 5). Relative distance along the GBR was also an important predictor of *C. albimarginatus* occurrence (relative influence: 21%) with a higher probability of occurrence in the southern GBR, particularly at sites with higher algae and hard coral cover. Relative distance along the GBR was the best predictor for the occurrence of *G. cuvier* (relative influence: 31.5%) with individuals likely to be found in the southern GBR (relative distance across <0.3; Fig. 1, Fig 5). There was also a higher probability of occurrence in close proximity to reefs (<5 km) and in deeper waters (>80 m) (Fig. 5). *Triaenodon obesus* was most likely to occur near coral reefs (relative influence: 19.5%) and at sites with high soft-coral cover (14.3%). Both the relative distance along the GBR and hard coral cover had a combined relative influence of over 20% for *T. obesus* (Fig. 4).

### Evaluating the effect of zoning on shark abundance

The effect of zoning on shark abundance was examined at 154 sites (26% of all sites; 1,120 BRUVS) between 2006 and 2010 (Fig. S2a). The distribution of sites sampled in areas open and closed to fishing did not vary significantly (Kolmogorov-Smirnov test D = 0.098, P = 0.861). Sampled sites had similar coral cover (Fig. S2b; Kolmogorov-Smirnov test D = 0.169, p = 0.228), but reef proximity varied between fished and non-fished sites (Kolmogorov-Smirnov test D = 0.288, P = 0.004). Conversely, 48% of sites (open and closed to fishing) were sampled at distances <4 km from a reef (Fig. S2c). The relative abundance of all sharks combined (MaxN hr^−1^) varied significantly between fished (2.25±0.32) and non-fished sites (4.23±0.56) (t-test  = 3.06, df - = 132, P = 0.003; Fig. S3).

**Figure 6:**
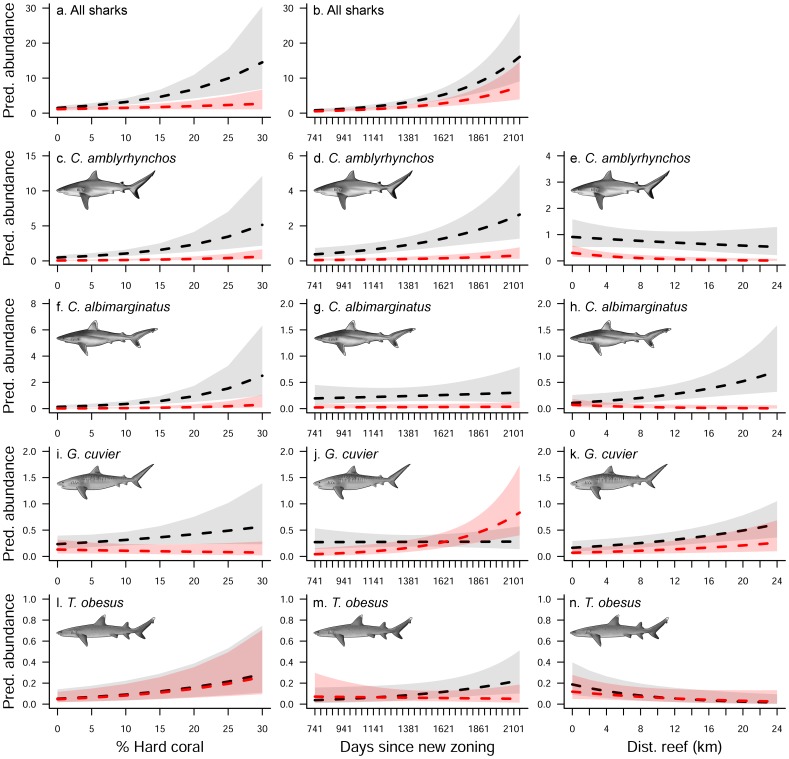
Effect of zoning on shark abundance, Great Barrier Reef of Australia. The predicted abundance for (a, b) all shark species pooled, *Carcharhinus amblyrhynchos* (c, d, e), *C. albimarginatus* (f, g, h), *Galeocerdo cuvier* (i, j, k), and *Triaenodon obesus* (l, m, n) was examined across the range of hard coral cover (%), days since the new zoning (effective since July 2004) and nearest distance to reef (km). Areas closed (black lines) and open (red lines) to fishing and 95% confidence intervals are shown.

Negative binomial and Poisson models were used to examine the effects of zoning, time (days since re-zoning) and habitat (hard coral cover and distance to reef) on shark abundances. The NB model had a better fit and lowest AIC value for all sharks combined (Table 3a). The best model did not include distance to reef or the interaction term (zoning × distance to reef), and fitted the data significantly better than the null model (i.e. the intercept-only model) (Likelihood test, P<0.0001). In this model, all individual predictors were statistically significant; however, there were no significant interactions. Shark abundances were significantly greater in areas closed to fishing, and the effect was significantly greater in sites with higher coral cover (Fig. 6a). In addition, the abundance of all sharks combined increased in both fished and non-fished sites with time, suggesting that since the 2004 re-zoning of the GBR some shark species have become more abundant (Fig. 6b).

**Table 3:** Summary results of Poisson (P) and negative binomial (NB) regression models used to examine the effect of zoning (areas closed/open to fishing) on the relative abundance of sharks (2004–2010).

Taxa	Terms	D.F	Deviance.	Residual D.F.	Resid. Dev	p-value
(a) All sharks - NB	Full model			153	287.54	
	Zoning	1	11.31	152	276.23	<0.001
	Days	1	93.41	151	182.83	<0.001
	Hard coral	1	21.49	150	161.33	<0.001
	Zoning × Hard coral	1	3.73	149	157.60	0.053
	Zoning × Days	1	0.20	148	157.40	0.650
(b) *C. amblyrhynchos* - NB	Full model			153	147.53	
	Zoning	1	14.34	152	133.19	<0.001
	Days	1	21.45	151	111.74	<0.001
	Hard coral	1	10.58	150	101.16	0.001
	Dist. reef	1	5.04	149	96.12	0.025
	Zoning × Hard coral	1	5.82	148	90.30	0.016
(c) *C. albimarginatus* - NB	Full model			153	128.78	
	Zoning	1	21.38	152	107.39	<0.001
	Days	1	0.74	151	106.66	0.391
	Hard coral	1	22.09	150	84.57	<0.001
	Dist. reef	1	1.86	149	82.70	0.172
	Zoning × Dist. reef	1	11.28	148	71.42	<0.001
(d) *G. cuvier* - P	Full model			153	144.01	
	Zoning	1	0.53	152	143.48	0.465
	Days	1	4.19	151	139.29	0.041
	Hard coral	1	5.24	150	134.05	0.022
	Dist. reef	1	12.94	149	121.11	<0.001
	Zoning × Hard coral	1	0.60	148	120.51	0.438
	Zoning × Days	1	8.97	147	111.54	0.003
(e) *T. obesus* - P	Full model			153	117.88	
	Zoning	1	7.72	152	110.15	0.005
	Days	1	5.49	151	104.66	0.019
	Hard coral	1	4.83	150	99.84	0.028
	Dist. reef	1	13.10	149	86.74	<0.001
	Zoning × Hard coral	1	2.15	148	84.59	0.143
	Zoning × Days	1	0.50	147	84.10	0.484

The performance of P and NB models were compared using Akaike's information criterion (AIC) against nested models and significant differences were evaluated with maximum likelihood ratio tests (χ^2^, p<0.05). Species: *Carcharhinus amblyrhynchos*, *C. albimarginatus*, *Galeocerdo cuvier* and *Triaenodon obesus*.

The effect of zoning was examined on three of the most common species sighted between 2006 and 2010. The negative binomial model performed better for *C. amblyrhynchos* and *C. albimarginatus*, whereas the Poisson model had a better fit and the lowest AIC value for *G. cuvier* and *T. obesus* (Likelihood test, P<0.0001; Table 3). For *C. amblyrhynchos*, the interaction between zoning and distance to reef was dropped from the model. All individual predictors were significant and there was a significant interaction effect of zoning × hard coral (Table 3b). A greater abundance of *C. amblyrhynchos* was observed in areas closed to fishing, which was influenced by both habitat and days since zoning. However, the overall effect of hard coral cover (Fig. 6c) was greater than the effect of time (Fig. 6d) and distance to reef (Fig. 6e). The abundance of *C. albimarginatus* was significantly greater on sites closed than open to fishing, particularly those that had high hard coral cover (Fig. 6f). There was no effect of days since zoning on the abundance of *C. albimarginatus* (Fig. 6g), however, there was a significant interaction between zoning and hard coral cover (Table 3c; Fig. 6h). The model predicted greater abundances of *C. albimarginatus* at sites that were farther from reefs, but only at non-fished sites (Fig. 6h). For *G. cuvier* the best fitting model included all possible predictors and their interactions (Table 3d) and the model showed an effect of hard coral cover (Fig. 6i), time (Fig. 6j) and distance to reef (Fig. 6k). The abundance of *G. cuvier* did not vary with zoning, however, there was a significant interaction effect between zoning and time (Table 3d). In areas open to fishing, *G. cuvier* abundance increased with time since zoning, while abundance remained the same in areas closed to fishing (Fig. 6j). Finally, the model showed that all the predictors had a significant effect on the abundance of *T. obesus*, but not the interactions (Table 3e). Higher abundances of *T. obesus* were observed at non-fished sites, especially those with high hard coral cover (Fig. 6l) and that were closer to reefs (Fig. 6n). There was also an increase in the abundance of *T. obesus* at non-fished sites with time (Fig. 6m).

## Discussion

Approximately 30% (21 species) of the total shark diversity reported for the entire GBRMP were sighted using BRUVS [14], [15]. However, the current study did not include all the available environments where sharks are known to occur. For example, ten species of shark that inhabit pelagic waters and twenty-eight occurring in bathyal/deep water (>200 m) habitats have been reported for the GBRMP [14]. BRUVS were restricted to relatively shallow habitats (<115 m) along the continental shelf, thus excluding pelagic and bathyal species. Therefore, when accounting for only shelf-water species, BRUVS were able to record >50% of the total shark diversity in nearshore and shelf habitats of the GBR.

Studies using different sampling methods have reported similar species richness, but different shark composition for the GBR (Fig. S4). For example, Harry et al. (2011) showed that the East Coast Inshore Finfish Fishery (ECIFF) operating within the GBR catches twenty-eight shark species. Although, the ECIFF is restricted to nearshore habitats [8], it shared at least seventeen shark species with BRUVS. The East Coast Trawl Fishery (ECTF) catches 38 species of sharks and rays, however, sharks occurred in relatively low numbers [51] and only seven of those species were observed during BRUVS surveys (Fig. S4). This could be due to a lack of interest in bait, preference for habitats that were not sampled consistently by BRUVS, or habitats that had low visibility during surveys. Seven shark species associated with the commercial Coral Reef Finfish Fishery (CRFF) [26] were also recorded by BRUVS. Interestingly, non-reef shark species were virtually absent from the CRFF [26], but BRUVS data included a large number of non-reef sharks species associated with coral reef habitats. Collectively, these studies suggest that while BRUVS recorded a large number of shark species, they may underestimate the occurrence of some species that seem to be more common in trawl and gill-net surveys. Therefore, using different sampling techniques simultaneously can improve estimates of shark species richness and composition.

### Shark assemblage structure

Most of the shark species observed using BRUVS have wide distributions and occupy diverse habitats, ranging from shallow coastal/inshore bays and estuaries, to inter-reefal shelf and coral reefs [8], [14], [15]. Contrary to other studies, depth was not a major factor predicting shark assemblages [2], [3]. Most shark species recorded in this study are highly mobile and use a wide range of available habitats [8], [9], [27]. Moreover, the GBR's continental shelf has relatively shallow depths [42], which may facilitate shark dispersal within and between different environments [9], [27], [52]. Detailed examination of BRUVS revealed that shark distribution patterns were mainly influenced by relative distances along and across the shelf and hard coral cover. In the northern GBR, coral reefs are typically closer to shore (<10 km), compared to central and southern regions (>100 km) [35], [53]. The distribution and density of the coral reef matrix along and across the GBR is likely to influence the occurrence of reef-associated species [35]. This study showed a higher probability of shark occurrences in the southernmost and northernmost sites of the GBR, while shark sightings decreased within the central region. A similar, but less prominent pattern was observed for shark species richness. Some sites south of Mackay (e.g. Swains and the Capricorn Bunker Group) and north of Cooktown (12–14.5°S) had disproportionally high shark diversity. Similar findings have been reported for other groups of fishes along the GBR [35].

Over 95% of shark species recorded by BRUVS were sighted at or near (<5 km) reef habitats, highlighting the importance of coral reefs for a large number of shark species throughout the GBR. In the narrow, northern GBR shelf, the higher density of reefs and proximity of surveyed sites to coastal bays and estuaries may have increased the number of shark sightings, and thus estimates of diversity. The remaining species recorded were mainly associated with non-reef habitats, characterized by soft-sediment substrates, from inshore bays/mangrove estuaries to the deeper continental shelf. Although coral reefs comprise only 5–6% of the habitats available in the GBR [53], our results showed a large number of sharks occurred near reef habitats. Coral reefs have been studied more intensively than other habitats as they: 1) are easy to access; 2) have a high structural complexity; 3) are among the most productive ecosystems on the planet; and 4) have disproportionately high biodiversity [13]. However, over the past few decades coral reefs have suffered declines in abundance, diversity, and structure, making them a high priority ecosystem for conservation [21], [54].

Reef-associated sharks include species that differ in size, life-history, and degree of association with coral reef habitats. Species like *T. obesus* and *C. amblyrhynchos* are known to spend most of their time on a single reef [6], [7], [30], whereas as other species (e.g. *G. cuvier*, *Sphyrna mokarran*, *C. leucas*) are more mobile and use a wide range of habitats [10], [20], [55]. In the present study, *C. amblyrhynchos*, *C. albimarginatus*, *T. obesus* and *G. cuvier* were sighted in over 35% of the sites and accounted for over 60% MaxN. These four species were also identified as indicator species and are likely driving most of the patterns of shark assemblages with respect to the distribution of coral reef habitats along the GBR.

### Shark species-specific habitat associations

The importance of coral reefs for reef-resident sharks such as *C. amblyrhynchos* and *T. obesus* has been extensively documented [6], [30], [56]–[58]. Our study showed that although these species were distributed throughout the entire GBR, they were more commonly sighted near the Capricorn-Bunker Region (southern GBR: 20.5–24°S). Catch data from the CRFF revealed no differences in reef shark abundances throughout the GBR, however, catches of *C. amblyrhynchos* and *T. obesus* in the Capricorn-Bunker Region were higher than expected based on the amount of fishing effort [26], thus supporting our observations. Other species like *G. cuvier* and *C. albimarginatus* were also commonly sighted in reef habitats near the Swains and Capricorn Bunker Group, with fewer sightings north of Townsville. *Galeocerdo cuvier* is known to use a wide diversity of habitats, ranging from bays and estuaries [59], [60] to coral reefs [10], [61]–[63]. Recent studies have shown that while some *G. cuvier* are year-round reef residents [61], [62], other individuals use coral reefs opportunistically or seasonally for feeding and reproduction [10], [63]. Moreover, long-range movements (1,114 km) across the Coral Sea have been reported for *G. cuvier*, indicating that some individuals also undertake long-range dispersals across deeper habitats [10]. Little is known about the ecology of *C. albimarginatus* despite its wide distribution [14]. Data from four *C. albimarginatus* acoustically tagged at Osprey Reef (Coral Sea) suggested that some individuals were year round residents, whereas others appeared more mobile [30]. Our study demonstrated that *C*. *albimarginatus* is a numerically important reef-associated species, completely absent from inshore sites, and only observed at one site in the central and northern GBR. These results suggest that *C*. *albimarginatus* has a strong association with offshore habitats near the coral reef matrix. However, further studies are needed to elucidate patterns of habitat use and long-term residency on coral reefs.

Distance along the GBRMP was consistently identified as an important predictor for shark occurrence. However, this result needs to be interpreted with caution as the low probability of shark occurrence in the central and northern GBR may be due to sampling bias. Although BRUVS were deployed throughout the entire GBR, some of the southern sites were sampled more intensively to answer specific questions that were outside the scope of this study. This may have influenced observed distribution patterns of shark species with respect to the effects of latitude. To control this sampling bias, individual BRUVS were pooled by site (i.e. sites were sampled on different dates and shared similar habitat/environmental conditions) and presence/absence data were used in the analyses instead of abundance.

Contrary to the findings of [35], this study showed that the occurrence of indicator shark species decreased abruptly from southern to northern sites, with the highest probability of occurrence at southern sites between 20.5° and 24°S. Their results suggested that changes in the assemblage of marine vertebrates along the GBR were likely due to latitudinal gradients in flushing rates (e.g. rate at which the water within 20 km of the coast is flushed with outer lagoon water; [64]) and the range of seasonal variation in sea surface temperature (SST) and salinity. Salinities in the southern GBR lagoon are higher than in the central and northern regions, while seasonal changes are typically lower [65]. Moreover, the central and northern GBR lagoons are generally more productive, and thus these areas considered to be important for coastal and inshore fish communities [53]. Our data showed that SST and chlorophyll-a concentration had little influence on shark distribution and/or species richness. However, it is possible that other environmental variables such as water current may be an important driver of shark assemblages in the southern GBR. Data from the Seafloor Biodiversity Project showed that bottom water current was significantly higher in the southern GBR (Table S2; [66]). Many reef-associated species, including non-resident sharks, tend to form predictable aggregations in areas of greater structural complexity (e.g. seamounts, outer parts of reef slopes and crests) and strong current flow, which may offer suitable habitat and productive foraging grounds [55], [67], [68]. Therefore, water current may be a more important predictor of shark occurrence than some of the environmental variables used in this study.

There are some limitations with the use of BRUVS that need to be considered.

First, most BRUVS could not be deployed directly on coral reefs or inside reef lagoons due to logistical constraints, which may have underestimated the abundance of species that commonly use these habitats such as blacktip reef sharks *C. melanopterus* [69], [70]. Nevertheless, estimates of habitat cover based on reference images revealed a high proportion of coral cover and the presence of structurally complex habitats (e.g. seagrass beds, soft-sediment inter-reef habitats, and rocky shoals dominated by diverse groups of octocorals, including soft corals, sea fans, sea pens) near reef sites. Second, the small field of view of BRUVS may have underestimated the number of sharks abundances recorded. For example, diving observations have revealed that species like *C. amblyrhynchos* can dominate the bait for the full period of the BRUVS recording while conspecifics maintained their distance outside the viewing areas of the cameras, and thus were less likely to be sighted [71]. Third, the quality of video recordings from BRUVS is affected by environments with high turbidity/low visibility (e.g. inshore/coastal bays and estuaries), which may have underestimated common shark species in these areas [11], [36], [72]. Fourth, although shark reference images were examined and identified by experts in the field, correct identification of some species using only video footage can be difficult. Moreover, species such as *C. limbatus* and *C. tilstoni* are known to hybridize in northern and eastern Australia [73]. Therefore, for analyses, closely related species that could be misidentified were excluded, and/or potential hybrids were pooled together (<5% of the sharks recorded). Fifth, the probability of shark sightings can depend on the time of day, as some species exhibit diel changes in behaviour and activity [67], [74]. For example, [37] showed that *Sphyrna lewini* and *S. mokarran* were important in characterizing BRUVS samples at night. Therefore, the small number of night-time sets used in this study (<2%) may have underestimated species that are more active at night. Conversely, species that were commonly sighted in this study such as *C. amblyrhynchos* and *C. albimarginatus* are typically found on coral reefs at night [M. Espinoza unpubl. data], indicating that BRUVS also recorded species that exhibit diel patterns of occurrence. Lastly, the use of bait to attract shark species may be biased by the distance and direction of the odour plume [75]. Some species are more readily attracted to bait or can influence the behaviour of others [71], [76]. It is important to note that other sampling methods such as trawls, long-lines and diver-based surveys also have limitations. Detectability varies by species in all observation methods, and variability in detectability is almost never accounted for in species richness calculations. Although BRUVS provide an ideal “non-destructive/non-extractive” approach for quantifying shark occurrences and species richness, combining different techniques may be more appropriate to fully define shark assemblages.

### Evaluating the effect of zoning on shark abundance

Within the GBRMP, there are several fisheries (e.g. ECIFF, ECTF, CRFF) that interact with sharks [8], [26], [51]. Most of the shark catch from the ECIFF is comprised of coastal/inshore species (e.g. blacktip *C. limbatus*/*C. tilstoni* and spot-tail *C. sorrah* sharks account for 54.8% of the catch). The ECTF catches a relatively high number of demersal elasmobranchs as by-catch, of which the orange spotted catshark *Asymbolus rubiginosus* accounts for approximately 50% of the shark catch [51] (Fig. S4). These species were either underrepresented (<3% MaxN) or not recorded at all in this study (Fig. S4). However, BRUVS recorded a large number of species that also occur in these fisheries, including *L. macrorhinus* (9.3% MaxN) and *Sphyrna* spp. (6.7% MaxN) which were also common in this study (Fig. S4). The absence of commonly observed species from the ECIFF and ECTF may be due to species-specific habitat preferences, sampling in environments with low visibility, or general lack of interest in the bait from BRUVS. Harry et al. (2011) also suggested that moderate-sized species like *C. limbatus*/*C. tilstoni*, *C. sorrah* and *Sphyrna* spp. are a major component of the ECIFF because they are more susceptible to capture by nets. Therefore, gillnets and bottom trawl surveys may be more effective at sampling cryptic species or species that have a high probability of capture. *Carcharhinus amblyrhynchos* and *T. obesus*, two of the most common species recorded in this study comprised over 90% of the catch from the CRFF [26]. While *C. amblyrhynchos* and *T. obesus* are a major component of the CRFF, it is important to note that fishing pressure for reef-associated sharks is relatively low. There are no dedicated reef shark fisheries and species that do interact with commercial and recreational line fisheries are typically taken incidentally. Moreover, long-term data from the CRFF revealed no evidence of increase or decline in shark catch rates [26]. However, sharks that interact with line fisheries may break off before landing or are released bearing hooks and traces, and thus it is unclear what the level of cryptic mortality is for some of these species [77]. Some studies within the GBR have argued that reef sharks have already experienced large population declines [78]–[80], which has attracted considerable concern by managers.

This study demonstrated that shark abundances were significantly higher in non-fished sites, highlighting the conservation value of the GBRMP zoning for sharks. However, the magnitude of those differences varied considerably among species, suggesting that the effect of zoning was species-specific. For example, non-fished sites had a greater abundance of *C. amblyrhynchos* and *C. albimarginatus* than *G. cuvier* and *T. obesus*. Although this could be biased by the overall lower sightings and/or residency behaviour, it could also mean that factors other than zoning may be influencing population sizes. Several studies have found a significant effect of zoning on shark abundance [5], [26], [81]. For example, within the GBR, [26] showed that areas closed to fishing were effective at protecting a portion of the shark population from exploitation, particularly species with strong site attachment. However, studies by [78] and [79] suggest that no-take zones, which are more difficult to enforce than no-entry zones (<1% of the GBRMP), offer almost no protection for shark populations. In this study, only no-take zones were considered in the analyses, which shows that even no-take zones can afford protection for reef-associated sharks by reducing their exposure to fisheries.

Hard coral cover and reef proximity affected shark abundances, particularly at non-fished sites. However, the effects of habitat on MPA studies have been largely neglected [82], and therefore, conclusions about the benefits of MPAs for sharks may be driven by habitat quality rather than the actual effect of zoning. For example, a recent review by [82] showed that over 50% of MPA studies examined did not account statistically for habitat effects. By including both habitat and time since the 2004 GBR re-zoning a better understanding of the effect and benefits of zoning for sharks was defined. Zoning comparisons were also restricted to sites that had been historically open to fishing (before re-zoning), and thus controlled for confounding factors such as comparison of sites with differing lengths of closure.

The frequency of disturbances such as tropical cyclones, coral predation by crown-of-thorns starfish, and coral bleaching events have resulted in a 50% decline of coral cover within the GBR over the past two decades [21]. This is concerning as our results showed that hard coral cover had a significant effect on the abundance of reef-associated sharks at non-fished sites while the effect of time was variable, suggesting that coral cover may be an important driver in the success of MPAs. Conversely, removal of reef sharks can have an impact that propagates down the food chain (e.g. mesopredators release), may alter the numbers of primary producers, and ultimately loss of coral cover [32]. Therefore, declines of reef-associated sharks can also have an effect on the health and resilience of coral reef communities.

Our results also showed that since the 2004 re-zoning of GBRMP, there has been an increase in the abundance of some species, including *C. amblyrhynchos* and to some extent *T. obesus*. Although still early, this finding suggests that the re-zoning of the GBRMP has already benefited some species of sharks. It also indicates that the zoning effect reported by [25] was not simply due to prior effects, in which only “good reefs” were closed to fishing. Time since re-zoning did not have an effect on the abundance of *C. albimarginatus.* We hypothesized that before the re-zoning of the GBRMP, the abundance of *C. albimarginatus* was already different between open and closed reefs, and has not increased despite zoning changes. Contrary to other reef species examined, the abundance of *C. albimarginatus* in areas closed to fishing decreased with increasing distance to reef. Collectively, these results suggest that while having a strong association with coral reefs *C. albimarginatus* may be less site attached, and thus the benefits of closed areas are not necessarily restricted to the proximity of a reef. For example, *C. albimarginatus* may be using inter-reefal habitats that provide some structure or abundant resources. Previous studies using BRUVS have identified important habitat features along the GBR (e.g. rocky shoals, macro-algae sea grass beds, soft- and hard-coral habitats) that were unknown or previously unmapped [53], [83]. Therefore, sites farther from reefs are not necessarily devoid of coral cover or some type of structural complexity. By using both reef proximity and hard coral cover in the models we were able to account for potentially unmapped habitat features that may be important features for reef-associated species.

Numerous studies have argued that large MPAs and/or reserve networks are essential for shark conservation [5], [30], [31], and less attention has been given to other management measures that may be more effective for some species [84]. While protecting reef habitats may be beneficial for sharks that spend a large amount of time on a single reef, the conservation value of coral reef MPAs for mobile sharks that use a wider range of habitats is unclear. Behavioural differences within and between species, as well as the ecological context in which a species exists can have important management implications. For example, movement patterns of sharks at remote and isolated reef atolls (self-contained environments) are likely to differ from more dense, semi-continuous reef environments such as the GBR [6], [9], [27], [30], [69]. Additionally, several shark species are thought to undertake long-range dispersals for reproduction or parturition [10], [85]–[87]. Consequently, movement information is still needed to make meaningful predictions about the benefits, long-term conservation value and effectiveness of MPAs. Additionally, it is important to note that besides no-take MPAs, the GBRMP is also complemented by a range of legislated fisheries management measures to conserve and sustain shark populations exposed to the gillnet, trawl and line fisheries of the region. These management measures include limited allocation of fishing licenses, a total allowable catch, maximum size limits, the declaration of no-take species, the requirement for landed fins to be accompanied by shark trunks, by-catch reduction devices, and improved reporting mechanisms [88]. Therefore, the GBRMP's zoning should not be viewed as the only management option for shark conservation.

BRUVS allowed quantification of shark species richness and occurrence for the entire GBR in areas where fishing is prohibited and/or visual surveys are restricted to shallow depths. However, to assess the full extent of shark assemblages within the GBR, the use of BRUVS may be complemented with fishery dependent and independent surveys. Given the lack of detailed ecological data for many shark species within the GBR, this study provided a valuable contribution to the understanding of species-specific habitat associations in response to a range of drivers. This study demonstrated that shark abundances were significantly higher in non-fished sites, highlighting the conservation value and benefits of the GBRMP zoning. However, our findings also showed that hard coral cover has a large effect on the abundance of reef-associated species, and thus may be an important driver in the effectiveness and success of coral reef MPAs. Therefore, predicting shark distribution patterns and understanding the drivers responsible for those patterns is essential for developing sound management and conservation approaches for sharks.

## Supporting Information

Figure S1 **A baited remote underwater video station showing details of the removable bait arm, plastic camera housing and pegs for placement of ballast on the frame (a).** Images of *Carcharhinus amblyrhynchos* (b), *C. albimarginatus* (c) and *Galeocerdo cuvier* (d) in the BRUVS field of view.
(DOCX)

Figure S2 (a) The number of sites sampled with baited remote underwater video stations across time (days since new zoning). (b) Frequency distribution of sampled sites according to hard coral cover (%). (c) Frequency distribution of sampled sites according to distance to reef (km). Data correspond to the sampling period between 2006 and 2010.
(DOCX)

Figure S3 **Relative abundance of sharks (MaxN hr^−1^) in closed and open fishing sites recorded by baited remote underwater video station, Great Barrier Reef (2006–2010).** Stars showed significant differences between zoning (t-test; p<0.05).
(DOCX)

Figure S4 **Shark species composition recorded using different sampling methods.** Species: *Carcharhinus amblyrhynchos*, *C. albimarginatus*, *Galeocerdo cuvier*, *Loxodon macrorhinus*, *Sphyrna* spp., *Nebrius ferrugineus*, *Triaenodon obesus*, *C. plumbeus*, *C. tilstoni/C.limbatus*, *C. dussumieri*, *Rhizoprionodon taylori*, *C. sorrah*, *R. acutus*, *C. macloti*, *C.brevipinna*, *Carcharhinus fitzroyensis*, *Asymbolus rubiginosus*, *A. analis*, *Figaro boardmani*, *Heterodontus galeatus*, *Heteroscyllium colcloughi*, *Mustelus walkeri*, *Orectolobus maculatus*, *Hydrolagus lemures*, *Atelomycterus marnkalha*, *Hemigaleus australiensis*, *Eucrossorhinus dasypogon*, *Chiloscyllium punctuatum*, *C. melanopterus* and *S. fasciatum*. Catch data was obtained from published studies [see 8,26,51] Vern diagram shows the total number of species shared between baited remote underwater video station (BRUVS) and other Queensland fisheries.
(DOCX)

Table S1 Summary of the results from the principal component analysis (PCA) of the six major habitat types. This analysis was performed the RDA function in the “vegan” library of R statistical package v.3.0.2 [49].
(DOCX)

Table S2 Summary of environmental data from the Seabed Biodiversity Project, Great Barrier Reef. Benthic stress is a measurement of bottom water current. N – Number of baited remote underwater stations. Data obtained from [66].
(DOCX)

Dataset S1 **Dataset of baited remote underwater video station deployed in the Great Barrier Reef, Australia.**
(CSV)
