# Supplementary material for: Quantifying Shark Distribution Patterns and Species-Habitat Associations: Implications of Marine Park Zoning
Source: PLoS One. 2014 Sep 10;9(9):e106885. doi: 10.1371/journal.pone.0106885 (PMC4160204; doi:10.1371/journal.pone.0106885)
Supplement: Figure S1 — A baited remote underwater video station showing details of the removable bait arm, plastic camera housing and pegs for placement of ballast on the frame (a). Images of Carcharhinus amblyrhynchos (b), C. albimarginatus (c) and Galeocerdo cuvier (d) in the BRUVS field of view. (DOCX) [file pone.0106885.s001.docx]

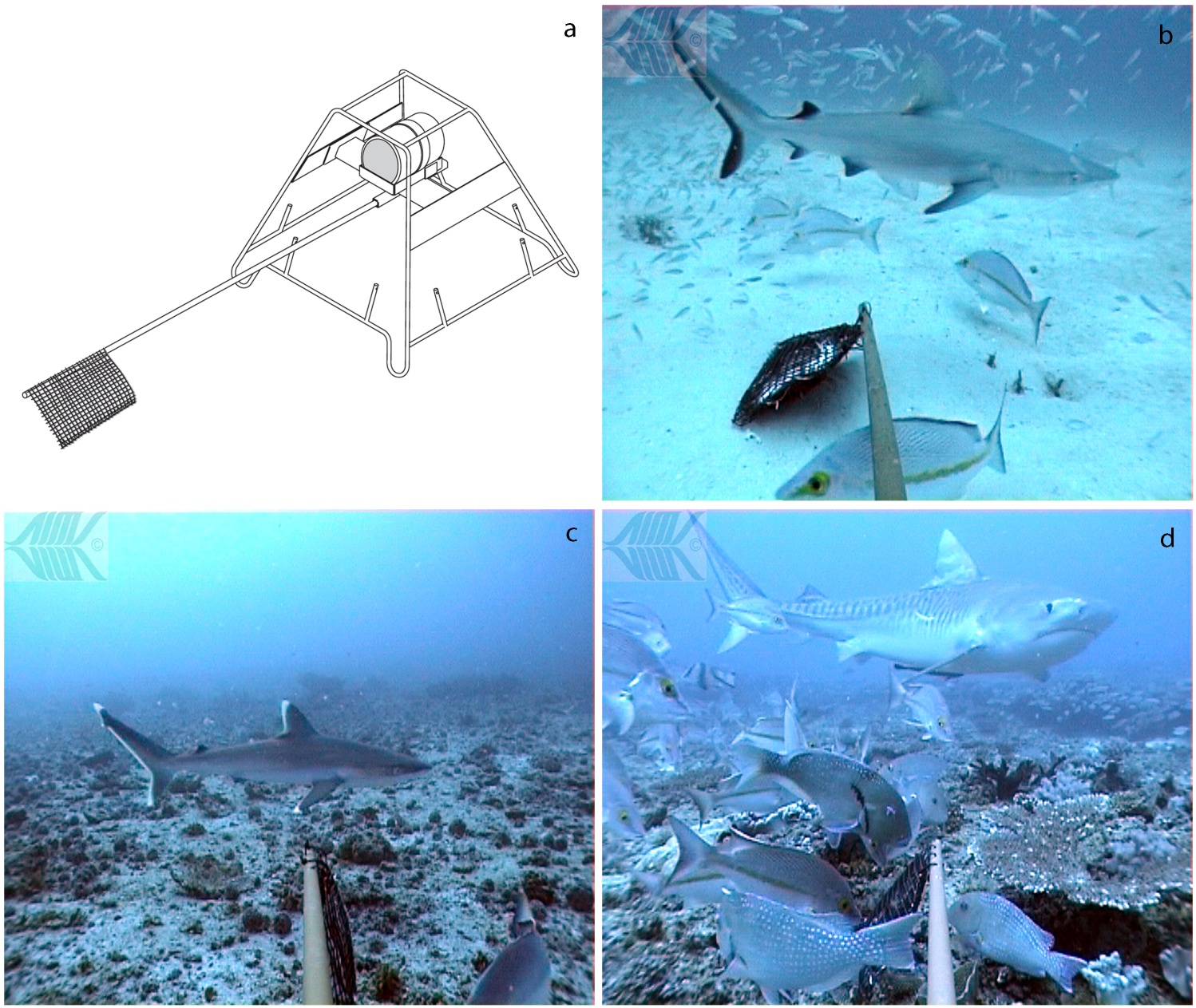


Figure S1 A baited remote underwater video station (BRUVS) showing details of the removable bait arm, plastic camera housing and pegs for placement of ballast on the frame (a). Images of *Carcharhinus amblyrhynchos* (b), *C. albimarginatus* (c) and *Galeocerdo cuvier* (d) in the BRUVS field of view.
