# Supplementary material for: Quantifying Shark Distribution Patterns and Species-Habitat Associations: Implications of Marine Park Zoning
Source: PLoS One. 2014 Sep 10;9(9):e106885. doi: 10.1371/journal.pone.0106885 (PMC4160204; doi:10.1371/journal.pone.0106885)
Supplement: Figure S2 — (a) The number of sites sampled with baited remote underwater video stations across time (days since new zoning). (b) Frequency distribution of sampled sites according to hard coral cover (%). (c) Frequency distribution of sampled sites according to distance to reef (km). Data correspond to the sampling period between 2006 and 2010. (DOCX) [file pone.0106885.s002.docx]

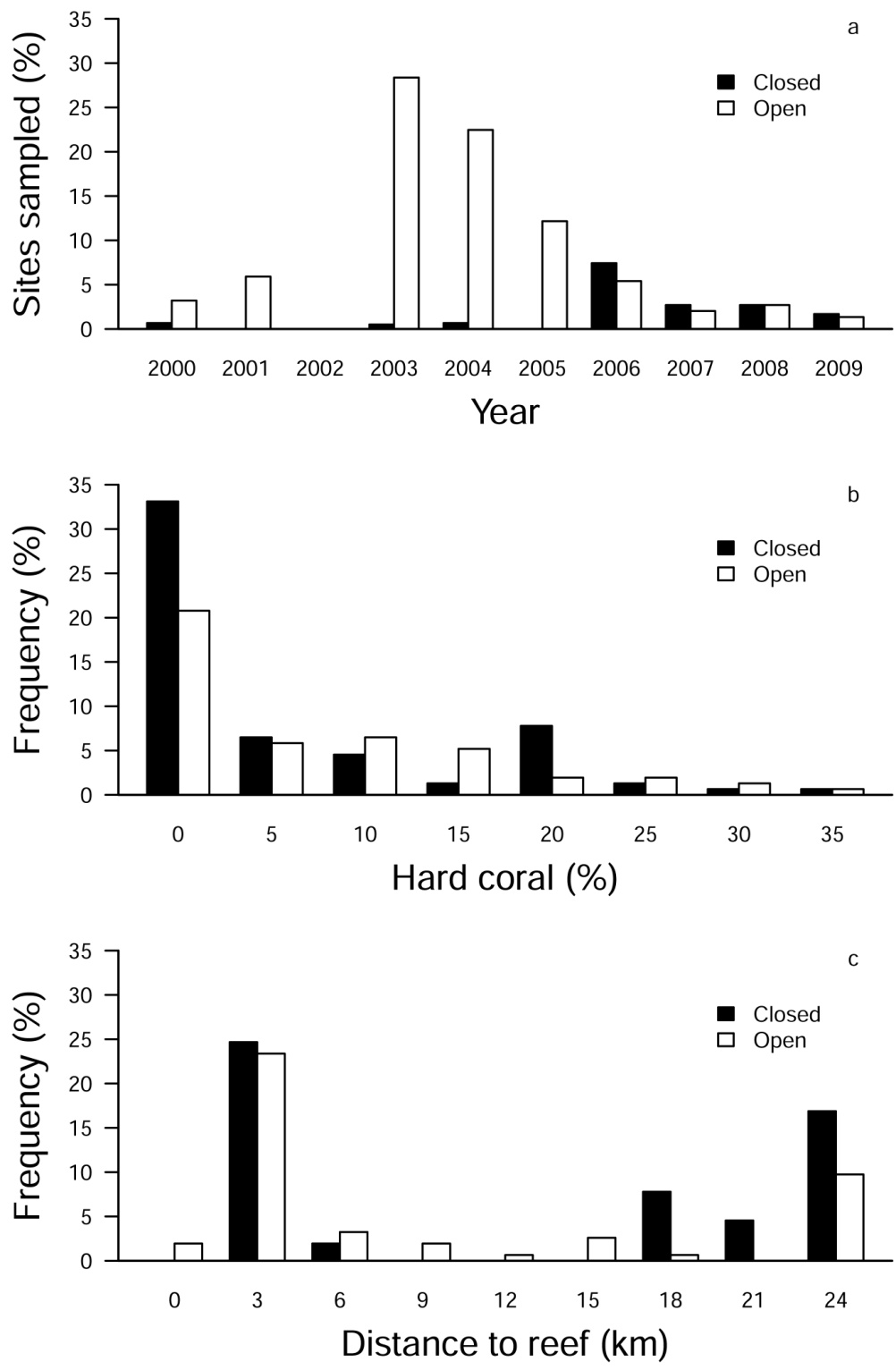


Figure S2. (a) The number of sites sampled with baited remote underwater video stations (BRUVS) across time (days since new zoning). (b) Frequency distribution of sampled sites according to hard coral cover (%). (c) Frequency distribution of sampled sites according to distance to reef (km). Data correspond to the sampling period between 2006 and 2010.
