# Supplementary material for: Quantifying Shark Distribution Patterns and Species-Habitat Associations: Implications of Marine Park Zoning
Source: PLoS One. 2014 Sep 10;9(9):e106885. doi: 10.1371/journal.pone.0106885 (PMC4160204; doi:10.1371/journal.pone.0106885)
Supplement: Figure S3 — Relative abundance of sharks (MaxN hr−1) in closed and open fishing sites recorded by baited remote underwater video station, Great Barrier Reef (2006–2010). Stars showed significant differences between zoning (t-test; p<0.05). (DOCX) [file pone.0106885.s003.docx]

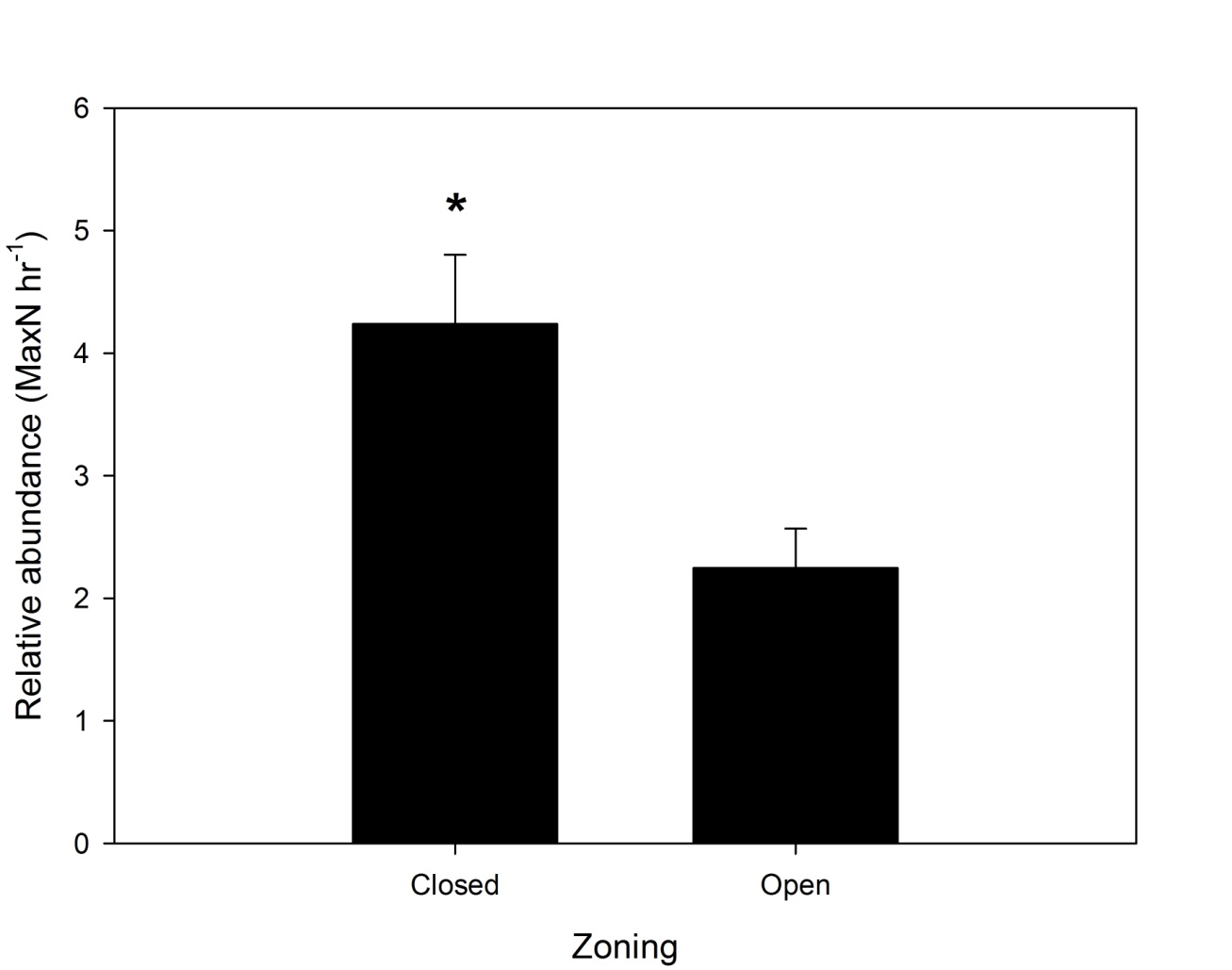


Figure S3. Relative abundance of sharks (MaxN hr^-1^) in closed and open fishing sites recorded by BRUVS, Great Barrier Reef (2006-2010). Stars showed significant differences between zoning (t-test; p < 0.05).
