# Supplementary material for: Quantifying Shark Distribution Patterns and Species-Habitat Associations: Implications of Marine Park Zoning
Source: PLoS One. 2014 Sep 10;9(9):e106885. doi: 10.1371/journal.pone.0106885 (PMC4160204; doi:10.1371/journal.pone.0106885)
Supplement: Figure S4 — Shark species composition recorded using different sampling methods. Species: Carcharhinus amblyrhynchos, C. albimarginatus, Galeocerdo cuvier, Loxodon macrorhinus, Sphyrna spp., Nebrius ferrugineus, Triaenodon obesus, C. plumbeus, C. tilstoni/C.limbatus, C. dussumieri, Rhizoprionodon taylori, C. sorrah, R. acutus, C. macloti, C.brevipinna, Carcharhinus fitzroyensis, Asymbolus rubiginosus, A. analis, Figaro boardmani, Heterodontus galeatus, Heteroscyllium colcloughi, Mustelus walkeri, Orectolobus maculatus, Hydrolagus lemures, Atelomycterus marnkalha, Hemigaleus australiensis, Eucrossorhinus dasypogon, Chiloscyllium punctuatum, C. melanopterus and S. fasciatum. Catch data was obtained from published studies [see 8,26,51] Vern diagram shows the total number of species shared between baited remote underwater video station (BRUVS) and other Queensland fisheries. (DOCX) [file pone.0106885.s004.docx]

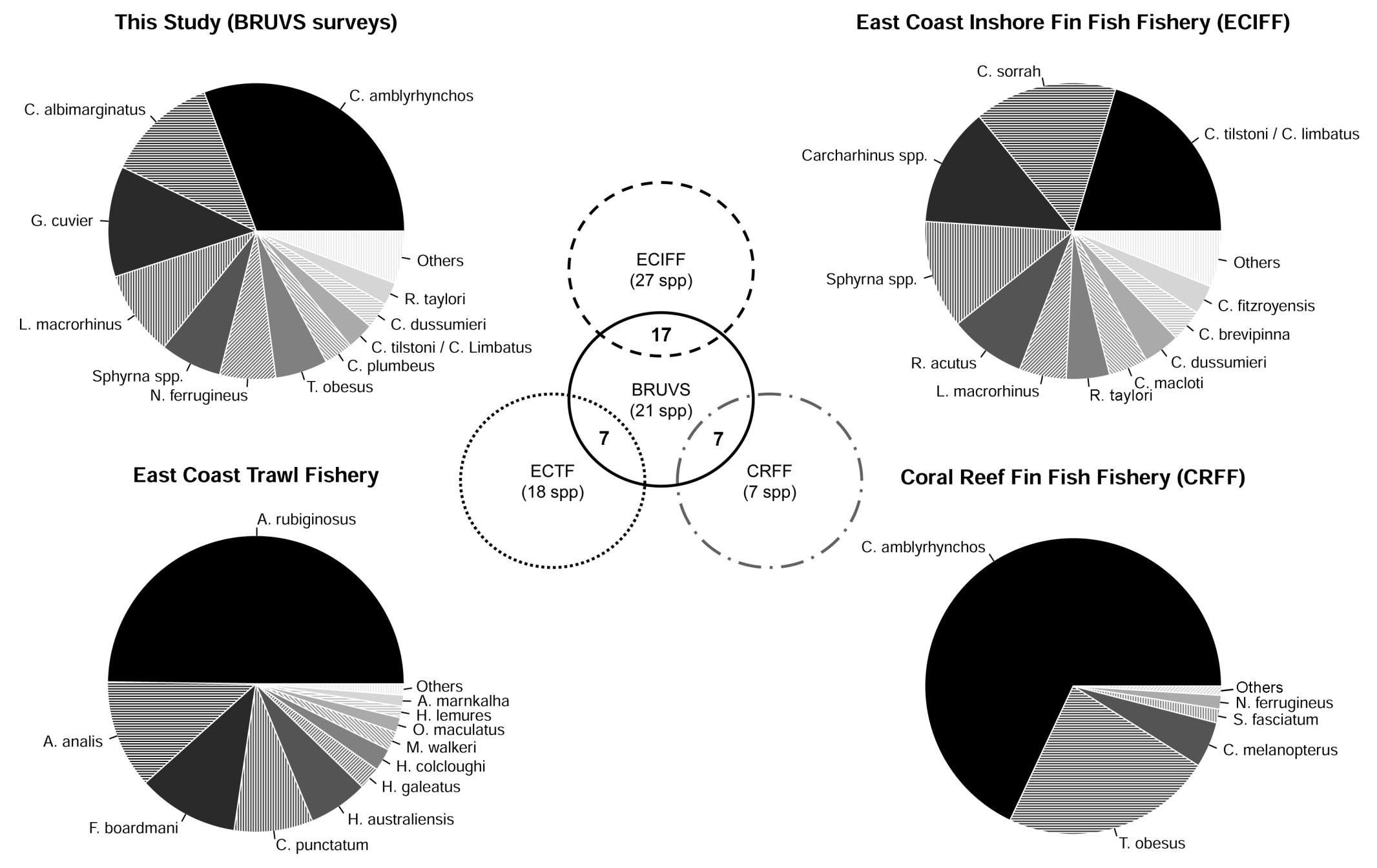


Figure S4. Comparison of shark species composition recorded using different sampling methods. Species: *Carcharhinus amblyrhynchos*, *C. albimarginatus*, *Galeocerdo cuvier*, *Loxodon macrorhinus*, *Sphyrna* spp., *Nebrius ferrugineus*, *Triaenodon obesus*, *C. plumbeus*, *C. tilstoni/C.limbatus*, *C. dussumieri*, *Rhizoprionodon taylori*, *C. sorrah*, *R. acutus*, *C. macloti*, *C.brevipinna*, *Carcharhinus fitzroyensis*, Asymbolus rubiginosus, A. analis, Figaro boardmani, Heterodontus galeatus, Heteroscyllium colcloughi, Mustelus walkeri, Orectolobus maculatus, Hydrolagus lemures, Atelomycterus marnkalha, *Hemigaleus australiensis*, *Eucrossorhinus dasypogon*, *Chiloscyllium punctuatum*, *C. melanopterus* and *S. fasciatum*. Catch data from the East Coast Inshore Fin Fish Fishery, the East Coast Trawl Fishery and the Coral Reef Fin Fish Fishery was obtained from published studies (see Kyne 2008, Heupel et al. 2009, Harry et al. 2011). Vern diagram shows the total number of species shared between BRUVS and different Queensland fisheries.
