# Supplementary material for: Quantifying Shark Distribution Patterns and Species-Habitat Associations: Implications of Marine Park Zoning
Source: PLoS One. 2014 Sep 10;9(9):e106885. doi: 10.1371/journal.pone.0106885 (PMC4160204; doi:10.1371/journal.pone.0106885)
Supplement: Table S1 — Summary of the results from the principal component analysis (PCA) of the six major habitat types. This analysis was performed the RDA function in the “vegan” library of R statistical package v.3.0.2 [49]. (DOCX) [file pone.0106885.s005.docx]

Table S1: Summary of the results from the principal component analysis (PCA) of the six major habitat types. This analysis was performed the RDA function in the "vegan" library of R statistical package v.3.0.2 (R Development Core Team 2008).

|  | PC1 | PC2 | PC3 |
| --- | --- | --- | --- |
| Eigenvalues | 1655.51 | 299.99 | 230.46 |
| Proportion explained | 0.726 | 0.132 | 0.101 |
| Cumulative proportion | 0.726 | 0.858 | 0.959 |
| Habitat scores |  |  |  |
| Algae/marine plants | -6.049 | 10.523 | -9.681 |
| Soft coral | -5.265 | 2.048 | -7.914 |
| Hard coral | -7.904 | 3.556 | 4.708 |
| Filter-feeders | -0.530 | 0.159 | -0.392 |
| Bare sand/mud | 36.233 | -3.146 | 0.704 |
| Encrusting algae/rubble | -16.485 | -13.140 | 1.213 |
