# Supplementary material for: Quantifying Shark Distribution Patterns and Species-Habitat Associations: Implications of Marine Park Zoning
Source: PLoS One. 2014 Sep 10;9(9):e106885. doi: 10.1371/journal.pone.0106885 (PMC4160204; doi:10.1371/journal.pone.0106885)
Supplement: Table S2 — Summary of environmental data from the Seabed Biodiversity Project, Great Barrier Reef. Benthic stress is a measurement of bottom water current. N – Number of baited remote underwater stations. Data obtained from [66]. (DOCX) [file pone.0106885.s006.docx]

Table S2. Summary of environmental data from the Seabed Biodiversity Project, Great Barrier Reef (GBR). Data obtained from Pitcher et al. (2009).

| Variable | South  (n = 161) | Central  (n = 138) | North  (n = 77) |
| --- | --- | --- | --- |
| Benthic stress (Pascals - N m^2^) |  |  |  |
| Range | 0.02 - 2.47 | 0.001 - 2.11 | 0.006 - 0.3 |
| Mean ± SD | 0.30 ± 0.34 | 0.18 ± 0.37 | 0.08 ± 0.08 |
| Temperature (°C) |  |  |  |
| Range | 13.98 - 9.89 | 9.89- 26.15 | 23.70 ± 1.78 |
| Mean ± SD | 23.69 ± 1.78 | 23.22 ± 3.43 | 23.01 ± 4.53 |
| Salinity (psu) |  |  |  |
| Range | 35.01 - 35.59 | 34.78 - 35.42 | 34.81 - 35.30 |
| Mean ± SD | 35.35 ± 0.11 | 35.16 ± 0.14 | 34.98 ± 0.11 |
| Dissolved oxygen (ml l^-1^) |  |  |  |
| Range | 4.03 - 5.05 | 3.59 - 4.75 | 3.4 - 4.63 |
| Mean ± SD | 4.61 ± 0.20 | 4.44 ± 0.24 | 4.34 ± 0.27 |
| Chlorophyll-a (mg m^3^) |  |  |  |
| Range | 0.21 - 3.3 | 0.14 - 7.45 | 0.31 - 3.09 |
| Mean ± SD | 0.63 ± 0.49 | 0.79 ± 1.02 | 0.79 ± 0.44 |
